# Optimal Salinity for Head-Starting Northern River Terrapins (*Batagur baska* Gray, 1831)

**DOI:** 10.3390/ani9110855

**Published:** 2019-10-23

**Authors:** Suthep Jualaong, Anida Songnui, Karun Thongprajukaew, Santi Ninwat, Suwandee Khwanmaung, Waraporn Hahor, Pairat Khunsaeng, Hirun Kanghae

**Affiliations:** 1 Marine and Coastal Resources Research and Development Center, Lower Gulf of Thailand, Songkhla 90100, Thailand; sutep.emcor@hotmail.com (S.J.); ninwat@hotmail.com (S.N.); 2 Trang Coastal Fisheries Research and Development Center, Trang 92150, Thailand; anida037@hotmail.com; 3 Department of Applied Science, Faculty of Science, Prince of Songkla University, Songkhla 90112, Thailand; tarn.something@gmail.com; 4 Satun Inland Aquaculture Research and Development Center, Satun 91110, Thailand; if-satuninland@hotmail.com; 5 Pattani Coastal Aquaculture Research and Development Center, Pattani 94150, Thailand; Pairatkhunsaeng@hotmail.com; 6 Phuket Marine Biological Center, Phuket 83000, Thailand; kanghae_H@hotmail.com

**Keywords:** carapace, digestive enzyme, elemental composition, feces, feed utilization, growth, head-starting program, hematological parameter

## Abstract

**Simple Summary:**

Northern river terrapins can adapt physiologically or behaviorally across a salinity gradient, so that they can move between freshwater habitats and brackish water or estuaries. However, there is no available information on the optimal salinity of this species for supporting captive husbandry programs before release to natural habitat. In the current study, the optimal salinity for the terrapins was investigated to serve the well-being of turtles. At the end of the rearing period, various observed parameters related to growth, feed utilization, and health indicated that optimal salinity for rearing the terrapins is about 4 ppt, while rearing in freshwater (0 ppt) or other brackish (8 and 12 ppt) water conditions had negative effects on some key parameters that reflect well-being. A polynomial regression fit to terrapin weight gain as function of salinity predicts the optimal salinity as 4.35 ppt. Findings from the current study could be directly used in ex situ conservation programs of northern river terrapins before release to natural habitat. Additionally, concurrent conservation and restoration of the wild habitats with preferred salinity is essential for flourishing terrapin population.

**Abstract:**

Northern river terrapins (*Batagur baska* Gray, 1831) are Asia’s largest turtles living in both freshwater and brackish water. In the current study, the optimal salinity for head-starting programs of this critically endangered species was investigated in order to serve the well-being of turtles before release to natural habitat. Forty-eight terrapins (54.64 ± 0.18 g initial body weight) were randomly distributed to four salinity levels (0, 4, 8, and 12 ppt) and reared for eight weeks, using three replicates with four terrapins each. At the end of rearing trial, growth performance and feed utilization parameters were superior in terrapins reared at 4 ppt, followed by 8 ppt in the rank order of treatments. Negative stress responses were observed in terrapins reared at 12 ppt, as the fecal activity of amylase-to-trypsin ratio was changed significantly, but not that of proteolytic enzymes. The fecal thermal transition properties indicated an abundance of nutrients in the post-absorptive phase for terrapins reared at 4 ppt, followed by the 8 ppt treatment group. The preferred 4 ppt salinity had no negative effects on the health status of the terrapins in terms of carapace elemental composition or hematological parameters. Second-order polynomial regression suggests 4.35 ppt as the optimal salinity for maximal weight gain. Findings from the current study could be directly used in ex situ conservation programs of northern river terrapins before release to natural habitat.

## 1. Introduction

Northern river terrapin (*Batagur baska* Gray, 1831) is Asia’s largest turtle, and is native to southeast Asian countries [1]. Due to consumption as food, this species is listed in the Convention on International Trade in Endangered Species of Wild Fauna and Flora (CITES), and is also classified as critically endangered by the International Union for Conservation of Nature and Natural Resources (IUCN). In order to conserve the population in the wild, captive breeding and hatchery projects were established in Vawal National Park in Bangladesh and at Sajnekhali Forest Station in India [1]. In Thailand, an ex situ head-starting program of this species before release to natural habitat has been conducted by the Department of Fisheries and the Department of Marine and Coastal Resources [2,3].

Northern river terrapins live in tidal brackish areas of the estuaries of medium and large rivers [4]; they can move between freshwater habitats and brackish water or estuaries. However, in published studies this species is commonly observed only in brackish water environment [5,6,7], while head-starting programs in Thailand are only conducted in freshwater [2]. These previous reports indicate that this species can adapt physiologically or behaviorally across a salinity gradient. However, weight loss after expose to various salinities (exceeding 17.5 ppt) has been reported for the species in genus *Batagur*, including *B. baska* and *B. borneoensis* [5,8]. Such prior data are not available specifically regarding the terrapins, so an investigation of the optimal salinity is essential for captive husbandry programs.

Feed utilization is also affected by the water salinity. In northern river terrapins, reduced feeding and drinking occurred when water salinity was too high [9]. These reductions are associated with a reduced metabolic rate due to the digestive activity, and are directly linked with reduced rate of excretion [10]. Therefore, understanding the mechanism for maintaining homeostasis under salinity stress can be tracked by the activities of digestive enzymes [11,12,13]. For endangered species like northern river terrapins, investigations of fecal digestive enzymes and fecal thermal properties are suitable for the non-invasive assessment of changes in endangered species such as green turtles, *Chelonia mydas* [14,15,16]. In addition, to improve the well-being in captive programs, the optimal salinity for head-starting northern river terrapins was investigated in the current study. The responses in fecal digestive enzymes, fecal thermal properties, carapace elemental profile, and hematological parameters were studied in non-lethal stress environments without conflict with ethical standards. The findings from the current study could be directly applied in pond or aquarium management for head-starting this endangered species. Concurrent conservation and restoration of wild habitats with the preferred salinity is essential for a flourishing terrapin population.

## 2. Materials and Methods

All use of animals conformed to the “Ethical Principles and Guidelines for the Use of Animals for Scientific Purposes”, National Research Council, Thailand (Application No. U1-06514-2560). Thirty-day-old terrapins were obtained from Satun Inland Aquaculture Research and Development Center, Satun, Thailand. The terrapins were acclimatized in a 500 L plastic tank (1 m diameter × 0.5 m depth, with 10 cm water level) for 5 days. They were fed ad libitum twice daily (at 9:00 and 15:00 h) with commercial floating feed pellet for tadpoles (Charoen Pokphand PCL., Samut Sakhon, Thailand) containing 32.50% crude protein. A 12-h light/12-h dark photoperiod was set for acclimatization. The water was renewed every day and 100% drained after feeding the second meal. At the end of the acclimatization period, 48 terrapins (54.64 ± 0.18 g initial body weight) were randomly distributed into four salinity levels (0, 4, 8, and 12 ppt) and reared for 8 weeks, with the procedures described above. The brackish water (4, 8, and 12 ppt) was prepared by diluting sea water with an appropriate freshwater ratio and was stocked for use until the end of experiment, while freshwater alone was used in the control treatment (0 ppt). The salinity levels were monitored using a reflecto-salinometer (S/mill-E; Atago, Tokyo, Japan). Four terrapins each were reared in twelve experimental units (i.e., glass aquarium—30 cm width × 60 cm length × 40 cm depth, with 10 cm water level). The water quality parameter ranges during the whole duration of experiment were: temperature 28.0 ± 1.1 °C (min–max: 25.2–31.1 °C), pH 7.57 ± 0.17 (7.09–7.84), alkalinity 50.50 ± 4.27 (40–60 mg L^−1^), and ammonia 0.07 ± 0.01 (0.05–0.08 mg L^−1^). Mortality was monitored daily over the whole duration of study. Body weight (BW), straight carapace width (SCW) and straight carapace length (SCL) were recorded every other week and are used for calculating weight gain (WG) and body condition index (BCI). Feeding rate (FR), feed conversion ratio (FCR), and protein efficiency ratio (PER) were estimated from the amount of feed pellets offered. At the end of the 8 weeks of trial, the terrapins were starved for 12 h prior to collecting samples for analysis, including samples of feces, carapace, and blood.

The pooled fresh feces (*n* = 3 per treatment) were quickly collected by dip net within one week before the end of experiment. The samples were carefully rinsed with cold distilled water three times to eliminate dirt, and then stored at –20 °C. The extraction was performed by mixing a sample with cold distilled water (1:10 *w*/*v*) and homogenizing in a micro-homogenizer (THP-220; Omni International, Kennesaw GA, USA). The homogenate was centrifuged at 15,000×*g* for 30 min at 4 °C and supernatant was collected, of which aliquots were kept at –20 °C until use. The concentration of protein (mg mL^−1^) in a crude enzyme extract was determined according to the standard method of Lowry et al. [17], using bovine serum albumin (BSA) as protein standard; these values were used to quantify the specific activity of enzymes (U mg protein^−1^).

All enzyme assays were performed within one month after extraction. The pepsin activity (EC 3.4.23.1) was assayed using hemoglobin as the substrate according to the method of Worthington [18]. The amount of liberated product was measured spectrophotometrically at 280 nm. One unit (U) of pepsin activity was defined as 1.0 increase in absorbance at 280 nm. The trypsin (EC 3.4.21.4) and chymotrypsin (EC 3.4.21.1) activities were assayed using *N*-benzoyl-*L*-Arg-*p*-nitroanilide (BAPNA) and *N*-succinyl-Ala-Ala-Pro-Phe-*p*-nitroanilide (SAPNA) as the substrates, respectively, according to Rungruangsak-Torrissen et al. [19]. The absorbance at 410 nm was measured and compared with a *p*-nitroanilide standard curve. The amylase activity (EC 3.2.1.1) was assayed using soluble starch as substrate based on Bernfeld [20]. The liberated product was quantified at 540 nm against a maltose standard curve. One unit (U) of trypsin, chymotrypsin, and amylase is defined as the amount that catalyzes the conversion of 1 μmol of substrate per minute.

The pooled feces (*n* = 3 per treatment) were dried using a freeze dryer (Delta 2-24 LSC, Martin Christ Gefriertrocknungsanlagen GmbH, Osterode am Harz, Germany) for 24 h to eliminate interference by water. The thermal transition properties of feces were measured in terms of onset (T_o_), peak (T_p_), and conclusion (T_c_) temperatures as well as enthalpy (∆H), using a differential scanning calorimeter (DSC7; Perkin Elmer, Waltham, MA, USA). Three milligrams of dried feces was placed in an aluminum pan and then heated from 40 to 400 °C at a rate of 10 °C min^−1^, while an empty pan was used as a reference. All peak temperatures and enthalpies were recorded by the DSC software.

The carapaces (*n* = 3 per treatment) were dissected (~ 1 mm × 1 mm) by aseptic scissors from the supracaudal scute [15] and then dried using a freeze dryer (Delta 2-24 LSC, Martin Christ Gefriertrocknungsanlagen GmbH, Osterode am Harz, Germany) for 24 h. The samples were mounted with two-sided adhesive tape on an aluminum stub and quantitative analysis of the elemental composition was carried out with a scanning electron microscope (Quanta 400; FEI, Brno, Czech Republic) equipped with an energy dispersive X-ray spectrometer (X-MAX, Oxford, UK). The accelerating voltage was set at 20 kV, and high vacuum mode and silicon drift detector (SDD) were used.

The terrapins were starved for 12 h prior to collecting the blood samples from dorsal cervical sinus (*n* = 3 pooled samples per treatment). All parameters were determined within 12 h after collection. Red (RBC) and white (WBC) blood cell counts from diluted samples were determined based on the method of Blaxhall and Daisley [21]. The packed cell volume (hematocrit) was determined according to the method of Larsen and Snieszko [22]. Mean cell volume (MCV) was calculated as described by Dacie and Lewis [23]. Differential leukocytes were counted from dried blood smears after fixing with methanol and staining with Jenner-Giemsa. Total and plasma proteins were determined based on the method of Lowry et al. [17]. Blood urea nitrogen (BUN), creatinine, alkaline phosphatase (ALP), alanine transaminase (ALT), and aspartate aminotransferase (AST) were determined using a commercial diagnostic kit (PZ Cormay S.A. Company, Lomianki, Poland). Serum cortisol from the untreated blood samples was determined by National Healthcare Systems Co., Ltd., accredited according to ISO 15189, based on electrochemiluminescence immunoassay (ECLIA).

A completely randomized design (CRD) was adopted, comprising four treatments with three replications having four terrapins each. All statistical evaluations were conducted in Statistical Package for the Social Sciences Version 14 (SPSS Inc., Chicago, USA). Arc sine transformation was applied to percentages prior to analysis. All data are reported as mean and standard error of mean (SEM). One-way ANOVA was used, and the mean comparisons were carried out using Duncan’s multiple range test at significance level α = 0.05 (*p* < 0.05). Second-order polynomials gave the best regression fit to WG as function of salinity, and this model gave an estimate of the optimal salinity. Indicators of growth and feed utilization were estimated as follows: survival (%) = (final terrapin number/initial terrapin number) × 100%; weight gain (g) = final weight (g) − initial weight (g); body condition index (BCI, kg cm^−3^) = (BW (kg)/SCL (cm)^3^) × 10^4^; FR (% BW day^−1^) = C/((W_0_ + W_t_)/2)/t × 100%, where C = daily feed consumption (g), W_0_ = initial body weight (g), W_t_ = final body weight (g), t = feeding duration (day); FCR (g feed g gain^−1^) = dry feed consumed (g)/wet weight gain (g); PER (g gain g protein^−1^) = wet weight gain (g)/protein intake (g)

## 3. Results

### 3.1. Survival, Growth Performance, and Feed Utilization

**Figure 1:**
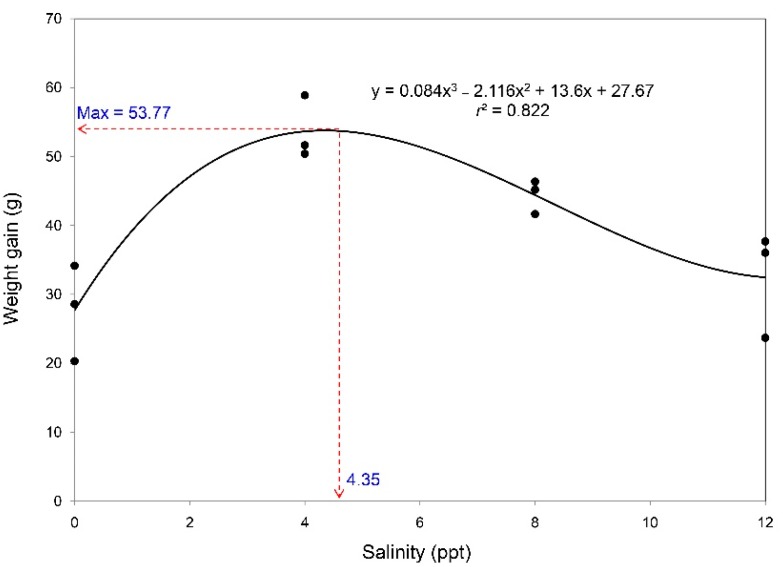
A polynomial regression fit to salinity level affecting the weight gain of northern river terrapins by the end of an eight-week experiment. Each point represents the average from three replicate groups of four subjects.

No mortality was observed during the eight weeks of experiment (Table 1). Final body weight and WG were superior in terrapins reared at 4 or 8 ppt over the other treatments (*p* < 0.05), while no differences between treatments were observed in SCW, SCL, SCL/SCW, or BCI. The terrapins reared at 4 ppt exhibited superior feed utilization, as indicated by comparatively low FCR and high FR and PER among the alternative treatments. Based on second-order polynomial regression of WG to salinity level, the predicted optimal salinity was 4.35 ppt (Figure 1).

### 3.2. Specific Activities of Fecal Digestive Enzymes

**Figure 2:**
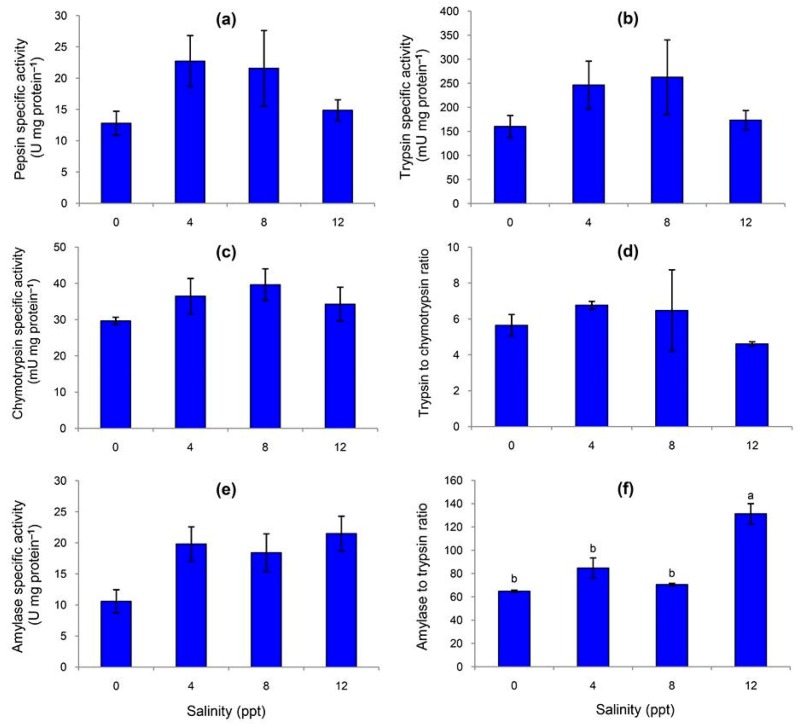
Specific activities of fecal digestive enzymes in northern river terrapins reared in various salinities: pepsin (**a**), trypsin (**b**), chymotrypsin (**c**), trypsin to chymotrypsin ratio (**d**), amylase (**e**), and amylase to trypsin ratio (**f**). The feces were sampled at the end of the eight-week experiment. Data are expressed as mean ± SEM (*n* = 3 per treatment). Different superscripts (a and b) indicate a significant difference (*p* < 0.05).

No differences in the specific activities of pepsin (Figure 2a), trypsin (Figure 2b), chymotrypsin (Figure 2c), trypsin to chymotrypsin ratio (T/C ratio, Figure 2d), or amylase (Figure 2e) were observed across the four salinity treatments. The terrapins reared at 12 ppt exhibited the highest amylase to trypsin ratio (A/T ratio), differing from the three remaining treatments (Figure 2f).

### 3.3. Thermal Properties of Feces

Dramatic differences in the fecal thermal properties (T_o_, T_p_, T_c_, T_c_–T_o_, and ΔH) were observed in terrapins subjected to the various salinities (Table 2). Rearing terrapins in freshwater caused absence of peak 1 in feces, while the brackish conditions did not give peak 3. Generally, the last three treatments were similar to the control treatment in thermal characteristics, except for T_c_–T_o_ and ΔH. The overall ΔH was highest for feces of terrapins reared at 4 ppt, followed by 8 and 12 ppt and control treatment in this rank order.

### 3.4. Elemental Composition in Carapace

Nine chemical elements were observed in the carapace samples of northern river terrapins, but seven of these (carbon, nitrogen, aluminum, phosphorus, sodium, chlorine, and magnesium) showed no differences between treatments (Table 3). Significantly decreased oxygen was observed in terrapins reared with 8 ppt salinity relative to the control freshwater treatment. The difference in sulfur contents was opposite to that for oxygen between these two treatments.

### 3.5. Hematological Parameters

RBC, hematocrit, MCV, WBC, azurophil, total protein, plasma protein, and AST did not differ across the four treatments (Table 4). Lymphocyte and BUN increased in a salinity-dependent manner, while thrombocytes and ALP decreased. The terrapins reared at 4 ppt had comparatively high heterophils and monocytes, while the other treatments gave comparatively low values in at least one of these parameters. Creatinine, ALT, and cortisol concentrations were very low across the five treatments—below the respective detection limits (<0.5 mg L^−1^, <5 U L^−1^, and <0.02 μg dL^−1^).

## 4. Discussion

Superior growth performance in the current study was observed for terrapins reared at 4 and 8 ppt and lesser performance was seen at 12 ppt. Within the same genus, weight losses for *B. baska* and *B. borneoensis* were reported at salinities above 17.5 ppt [5,8]. For maximal growth, similar results have been reported for some species, such as diamondback terrapin (*Malaclemys terrapin terrapin*): optimal salinity range is from 1.5 to 8.0 ppt while 12 and 16 ppt give poorer growth [24]; and 25% seawater (~ 9 ppt) is preferable to 0%, 50%, and 100% replacement of seawater by freshwater [25]; or 10 ppt is preferable to 0, 20, or 30 ppt [26]. However, based on feed utilization in the current study, salinity at 8 ppt was not optimal for this species since FR was inferior. This observation is in agreement with the response of *B. baska*, which experiences reduced feeding at high salinity levels [9]. FCR and PER of terrapins reared at 8 ppt were similar to the preferred treatment (4 ppt), suggesting that this species can adapt physiologically across this range of salinity and maintain feed utilization homeostasis. Therefore, freshwater turtles can be temporarily observed in marine or brackish environments [27]. Northern river terrapins live in tidal brackish areas of the estuaries of medium and large rivers [4], identifying as index III of saline habitat occurrence since they is common in brackish environments within and among publications [27]. Based on our observations of WG following a second-order polynomial response to salinity, the optimal salinity would be around 4.35 ppt. This preferred salinity is quite similar to that reported for red-eared slider (*Trachemys scripta elegans*): 5 ppt is preferred over 0, 15, 25, and 35 ppt [12].

Some large differences in WG were observed across the salinity treatments, while carapace dimension (SCW, SCL, and SCL/SCW) showed no differences, suggesting normal morphometric changes of carapace. However, after standardizing with SCL, the body morphometrics were still similar, as indicated by BCI. These indicate terrapin bodies are encased in a “box” made up of the carapace, plastron, and the carapace–plastron bridges. 

Variation in the salinity of the gastric lumen can cause significant changes in digestive enzyme activities for balancing energy demand in continuous ionic regulation [11,28]. Salinity level had no effects on the specific activities of pepsin, trypsin, chymotrypsin, or T/C ratio, suggesting that protein digestion in stomach and intestine operated normally. Similar results were also observed for specific activity of amylase for digesting carbohydrates in intestine. On the other hand, the A/T ratio was significantly affected by rearing at the highest salinity. A similar finding was also reported for juvenile hybrid grouper (*Epinephelus coioides* × *Epinephelus lanceolatus*) when reared at 30 ppt in comparison to 10, 15, and 20 ppt [13]. Since this digestive parameter is associated with carbohydrate utilization [29,30], the increased values indicate a high capacity of terrapins to utilize carbohydrates per amount of protein. However, the energetic cost of osmoregulation is higher in hypersaline treatment, in which the ion concentrations of blood and water are disparate, potentially making less energy available for growth. Therefore, when the turtles are subjected to stress from salinity, the blood glucose quickly increases, as reported by Shu et al. [31] and Hong et al. [32].

Physiological changes reflecting feed utilization were assessed via the thermal properties of feces in the current study. This technique has been used in our previous studies on endangered animals, as a non-invasive method is needed [14,15,33]. Comparative differences between rearing terrapins in fresh or brackish water were clearly observed, along with the four peaks indicating remaining available nutrients (low temperature, peaks 1 and 2) and unavailable nutrients (high temperature, peaks 3 and 4) present in the feces. Terrapins reared in the preferred condition (4 ppt) had the highest ΣΔH, indicating abundant native nutrients in the feces [16] or that the nutrients in excretions would be easily available; they would require more energy for transformation during heating. For terrapins reared at higher salinities, although improved ΣΔH was observed the FR was significantly decreased, leading to inferior performance. Similarly, unfavorable fecal thermal properties could be associated with significantly retarded FCR and PER in the control treatment. Overall, the findings from the current study also indicate changes in molecular properties of nutrients, since T_c_–T_o_ is associated with the heterogeneity of cleaved polymers after digestion [34].

Among the nine chemical elements observed in carapace, significant concentration differences were prominent only in oxygen and sulfur. Oxygen is a major element in various chemical compounds [15], and its replacement with sulfur was observed in terrapins reared at 8 ppt. The increased sulfur might be linked to the sulfur-containing amino acid cysteine in the structure of keratins of the carapace [35,36]. These proteins are associated with osmotic stress response to various salinities [37]. Understanding the mechanisms involved in the physiological responses could be pursued in further studies.

Various external factors, such as temperature, light, water quality, salinity, and other stress inducers, can greatly influence the immune system and incur responses in the reared animals [38]. Some changes in hematological parameters associated with salinity were observed in the current study. In broad-nosed pipefish (*Syngnathus typhle*), increased activity and proliferation of immune cells were observed after exposure to various salinities, but low salinity did not boost specific immune responses in monocyte and lymphocyte cell proliferation [39]. Significant changes in lymphocytes, heterophils, monocytes, and thrombocytes indicate negative effects from rearing terrapins at high salinity. BUN significantly increased with water salinity. This result is in agreement with data on red-eared slider reared at 5, 15, and 25 ppt [32]. Possible reasons might include increased urea retention in urinary bladder [40] or increased rate of urea synthesis [41]. The activity of ALP decreased with water salinity, while the activities of ALT and AST showed no negative changes. Generally, ALP participates in the degradation of foreign proteins, carbohydrates, and lipids [42]. No changes in ALP activity have been reported in juvenile tongue sole, *Cynoglossus semilaevis* [43] or blood parrotfish, *Cichlasoma synspilum* × *Cichlasoma citrinellum* [44] from exposure to various water salinities. Some species, such as cobia (*Rachycentron canadum*), have increased ALP activity when the salinity is within the range from 5 to 37 ppt [45]. These findings indicate that the strategy to acclimatize to a water environment is species specific. The plasma cortisol as indicator of stress response had concentration below the detection limit, <0.02 μg dL^−1^. This concentration of cortisol in terrapins is low compared to prior reports on some healthy tortoises or turtles [46,47]. The current study could be improved upon by more sensitive determination of this stress indicator parameter.

In conclusion, optimizing the salinity level for head-starting northern river terrapins was investigated in the current study. Various observed parameters related to growth, feed utilization (including fecal digestive enzyme activities and fecal thermal properties), and health (hematological parameters and carapace elemental profile) indicated that the optimal salinity for rearing the terrapins is about 4 ppt, while rearing in freshwater or other brackish water conditions had negative effects on some key parameters that reflect the well-being of terrapins. The polynomial regression fit to terrapin WG as function of salinity predicts the optimal salinity as 4.35 ppt. Findings from the current study could significantly impact the practiced conditions in head-starting northern river terrapins in Thailand, since previously they have been reared in freshwater.

## Figures and Tables

**Table 1:** Survival, growth performance, and feed utilization of northern river terrapins reared in various salinities. The observed parameters were recorded at the end of the 8-week experiment.

Parameter	Salinity (ppt)				*p*-Value
	0	4	8	12	
Survival (%)	100	100	100	100	–
Initial body weight (g)	54.03 ± 0.48	54.73 ± 0.26	55.20 ± 0.12	54.59 ± 0.22	0.121
Final body weight (g)	81.71 ± 3.71^b^	108.37 ± 2.70^a^	99.62 ± 1.51^a^	87.05 ± 4.30^b^	0.002
WG (g)	27.68 ± 4.02^b^	53.64 ± 2.65^a^	44.40 ± 1.42^a^	32.46 ± 4.41^b^	0.002
SCL (cm)	8.02 ± 0.21	8.67 ± 0.15	8.27 ± 0.08	7.99 ± 0.20	0.073
SCW (cm)	8.17 ± 0.17	8.66 ± 0.12	8.34 ± 0.07	8.16 ± 0.22	0.165
SCL/SCW	0.98 ± 0.01	1.00 ± 0.01	0.99 ± 0.01	0.98 ± 0.01	0.062
BCI (kg cm^−^^3^)	1.59 ± 0.06	1.66 ± 0.05	1.76 ± 0.02	1.71 ± 0.05	0.135
FR (% BW day^−1^)	0.67 ± 0.02^a^	0.69 ± 0.01^a^	0.59 ± 0.01^b^	0.54 ± 0.03^b^	0.002
FCR (g feed g gain^−1^)	0.90 ± 0.10^a^	0.55 ± 0.01^b^	0.54 ± 0.01^b^	0.64 ± 0.04^b^	0.004
PER (g gain g protein^−1^)	3.30 ± 0.33^b^	5.24 ± 0.13^a^	5.32 ± 0.03^a^	4.58 ± 0.26^a^	0.001

WG, weight gain; SCL, straight carapace length; SCW, straight carapace width; BCI, body condition index; FR, feeding rate; BW, body weight; FCR, feed conversion ratio, PER, protein efficiency ratio. Data are expressed as mean ± SEM (*n* = 12 per treatment). Differences between means were tested with Duncan’s multiple range test. Different superscripts (a and b) in the same row indicate a significant difference (*p* < 0.05).

**Table 2:** The thermal transition characteristics of feces of northern river terrapin reared in various salinities. The sampling was done at the end of the 8-week experiment.

Thermal Parameter	Salinity (ppt)			
	0	4	8	12
Peak 1				
T_o_ (°C)	-	43.67	44.29	47.97
T_p_ (°C)	-	64.61	67.45	63.44
T_c_ (°C)	-	81.17	102.25	79.64
T_c_–T_o_ (°C)	-	37.50	57.96	31.67
ΔH_1_ (J g^−1^)	-	18.57	27.06	21.86
Peak 2				
T_o_ (°C)	88.11	91.63	91.82	91.56
T_p_ (°C)	95.61	99.45	123.16	99.53
T_c_ (°C)	101.99	105.64	133.19	107.93
T_c_ – T_o_ (°C)	13.88	14.01	41.37	16.37
ΔH_2_ (J g^−1^)	2.81	2.91	3.01	2.07
Peak 3				
T_o_ (°C)	177.23	-	-	-
T_p_ (°C)	186.10	-	-	-
T_c_ (°C)	191.06	-	-	-
T_c_ – T_o_ (°C)	13.83	-	-	-
ΔH_3_ (J g^−1^)	4.93	-	-	-
Peak 4				
T_o_ (°C)	353.13	323.38	315.15	334.68
T_p_ (°C)	368.33	351.39	349.00	352.72
T_c_ (°C)	388.50	378.59	375.95	373.80
T_c_ – T_o_ (°C)	35.37	55.21	60.80	39.12
ΔH_4_ (J g^−1^)	23.32	86.11	28.21	20.20
ΣΔH (J g^−1^)	31.06	108.19	58.29	44.13

T_o_, onset temperature; T_p_, peak temperature; T_c_, conclusion temperature; ΔH, transition enthalpy; Data are expressed as means from triplicates.

**Table 3:** Carapace elemental profiles of northern river terrapin reared in various salinities. The sampling was done at the end of the 8-week experiment.

Element (% of dry weight)	Salinity (ppt)				*p*-Value
	0	4	8	12	
C	46.77 ± 0.80	48.38 ± 0.44	52.00 ± 1.99	47.16 ± 0.99	0.052
O	32.91 ± 0.58^a^	29.88 ± 0.48^ab^	27.63 ± 1.23^b^	30.56 ± 1.13^ab^	0.023
N	19.50 ± 0.28	21.19 ± 0.39	19.48 ± 1.35	21.91 ± 0.24	0.109
S	0.20 ± 0.03^b^	0.30 ± 0.01^b^	0.67 ± 0.17^a^	0.20 ± 0.02^b^	0.041
Al	0.23 ± 0.02	0.17 ± 0.05	0.13 ± 0.02	0.10 ± 0.02	0.085
P	0.10 ± 0.01	0.10 ± 0.01	0.10 ± 0.01	0.10 ± 0.01	1.000
Na	0.20 ± 0.06	0.10 ± 0.01	0.10 ± 0.02	0.17 ± 0.02	0.160
Cl	0.12 ± 0.02	0.12 ± 0.01	0.13 ± 0.03	0.10 ± 0.01	0.745
Mg	0.10 ± 0.01	0.10 ± 0.01	0.10 ± 0.01	0.10 ± 0.01	1.000

Data are expressed as mean ± SEM (*n* = 3 per treatment). Differences between means were tested with Duncan’s multiple range test. Different superscripts (a and b) in the same row indicate a significant difference (*p* < 0.05).

**Table 4:** Hematological parameters of northern river terrapin reared in various salinities. The sampling was done at the end of the 8-week experiment.

Hematological Parameter	Salinity (ppt)				*p*-Value
	0	4	8	12	
RBC (×10^6^ cells μL^−1^)	0.42 ± 0.04	0.43 ± 0.03	0.39 ± 0.04	0.37 ± 0.02	0.500
Hematocrit (%)	35.33 ± 1.76	38.00 ± 1.15	38.33 ± 2.33	36.33 ± 2.19	0.665
MCV (fL)	887.63 ± 59.75	879.68 ± 85.44	995.19 ± 40.32	995.55 ± 15.98	0.424
WBC (×10^3^ cells μL^−1^)	5.28 ± 0.11	5.83 ± 0.32	5.43 ± 0.62	3.36 ± 0.50	0.050
Lymphocyte (%)	27.33 ± 8.11^b^	31.00 ± 4.36^b^	56.00 ± 3.00^a^	57.67 ± 5.24^a^	0.015
Azurophil (%)	45.00 ± 9.00	53.00 ± 3.00	31.00 ± 2.00	35.67 ± 4.10	0.054
Heterophil (%)	4.00 ± 0.00^b^	7.00 ± 1.00^a^	1.50 ± 0.50^c^	2.00 ± 1.00^bc^	0.007
Monocyte (%)	15.33 ± 1.76^a^	9.33 ± 1.76^ab^	13.33 ± 2.33^a^	3.50 ± 0.50^b^	0.020
Thrombocyte (×10^3^)	2.06 ± 0.20^a^	1.45 ± 0.34^ab^	0.94 ± 0.06^bc^	0.56 ± 0.06^c^	0.023
Plasma protein (g %)	2.40 ± 0.12	2.80 ± 0.12	2.53 ± 0.33	2.60 ± 0.31	0.705
Total protein (g L^−1^)	17.63 ± 1.14	15.80 ± 1.25	13.43 ± 1.49	13.80 ± 0.70	0.115
BUN (mg dL^−1^)	22.87 ± 1.76^c^	32.94 ± 1.51^c^	57.85 ± 0.71^b^	159.03 ± 8.93^a^	<0.001
ALP (U L^−1^)	110.30 ± 0.90^a^	88.63 ± 0.52^b^	72.95 ± 3.95^c^	61.30 ± 4.60^d^	<0.001
AST (U L^−1^)	118.23 ± 5.69	110.45 ± 4.55	114.00 ± 14.90	118.60 ± 14.50	0.927

RBC, red blood cells; MCV, mean cell volume; WBC, white blood cells; BUN, blood urea nitrogen; ALP, alkaline phosphatase; AST, aspartate aminotransferase. Data are expressed as mean ± SEM (*n* = 3 per treatment). Differences between means were tested with Duncan’s multiple range test. Different superscripts (a, b, c and d) in the same row indicate a significant difference (*p* < 0.05).
